# NSSI questionnaires revisited: A data mining approach to shorten the NSSI questionnaires

**DOI:** 10.1371/journal.pone.0284588

**Published:** 2023-04-21

**Authors:** Nacer Farajzadeh, Nima Sadeghzadeh

**Affiliations:** 1 Faculty of Information Technology and Computer Engineering, Azarbaijan Shahid Madani University, Tabriz, Iran; 2 Artificial Intelligence and Machine Learning Research Laboratory, Azarbaijan Shahid Madani University, Tabriz, Iran; Sejong University, REPUBLIC OF KOREA

## Abstract

**Background and objective:**

Non-suicidal self-injury (NSSI) is a psychological disorder that the sufferer consciously damages their body tissues, often too severe that requires intensive care medicine. As some individuals hide their NSSI behaviors, other people can only identify them if they catch them while injuring, or via dedicated questionnaires. However, questionnaires are long and tedious to answer, thus the answers might be inconsistent. Hence, in this study for the first time, we abstracted a larger questionnaire (of 662 items in total) to own only 22 items (questions) via data mining techniques. Then, we trained several machine learning algorithms to classify individuals based on their answers into two classes.

**Methods:**

Data from 277 previously-questioned participants is used in several data mining methods to select features (questions) that highly represent NSSI, then 245 different people were asked to participate in an online test to validate those features via machine learning methods.

**Results:**

The highest accuracy and F1 score of the selected features–via the Genetics algorithm–are 80.0% and 74.8% respectively for a Random Forest algorithm. Cronbach’s alpha of the online test (validation on the selected features) is 0.82. Moreover, results suggest that an MLP can classify participants into two classes of NSSI Positive and NSSI Negative with 83.6% accuracy and 83.7% F1-score based on the answers to only 22 questions.

**Conclusion:**

While previously psychologists used many combined questionnaires to see whether someone is involved in NSSI, via various data mining methods, the present study showed that only 22 questions are enough to predict if someone is involved or not. Then different machine learning algorithms were utilized to classify participants based on their NSSI behaviors, among which, an MLP with 10 hidden layers had the best performance.

## Introduction

Non-suicidal self-injury (NSSI) is a psychological disorder that causes the sufferer to intentionally damage the tissues of their body, which are often severe and require intensive care medicine, without intending to be dead [1]. People of any age can be involved in this disorder; however, it is more prevalent in adolescents and youngsters [2]. It is said that the onset age of the involvement in the disorder usually varies from 12 to 14 [3–7]. Although NSSI does not intend to end one’s life, people who are involved in NSSI show a higher desire for committing suicide [5, 8] as their pain tolerance increases and they can be more ruthless with themselves [9]. Hence, NSSI is the second potential leading cause of death among people who are between 15 to 19 years old, and the 10th among those between 10 to 14 years old [10]. Clearly, this disorder has many negative impacts on the sufferers’ lifestyles, and therefore, identifying people who could be or become involved with NSSI can play an important role in controlling, treating, and preventing it in their older ages [11–13].

Although ample research has been conducted to identify people with NSSI planning and those without it, recent analyses show that our ability in doing so has been quite limited since only a few factors in people’s lives have been considered, not all of them [14]. Consequently, the prognosis of NSSI is still not accurate [15]. NSSI and suicidal behaviors can be differentiated by (but not limited to) nine major factors: intent, lethality, chronicity, methods and functions, cognitions, reactions, aftermath, demographics, and prevalence [5]. While NSSI is primarily intended to eliminate 1) negative emotion, 2) body alienation (dissatisfaction), and 3) dissociation, suicide tends to be a way out of all of them at once when the injurers are not satisfied with the deceptive temporary pain caused by injuries, nor are they cured via therapies [1, 5, 16–23].

NSSI is a complex concept due to various intertwined factors that contribute to its conception in one’s mind, and this complexity hinders psychologists from a clear understanding of the factor(s) they should fix in an injurer’s life [24, 25]. This makes it more likely that the majority of the injurers will still be involved in NSSI in different ways as no one can thoroughly take into consideration their needs and motivations [24, 25]. Researchers in this field have come up with a comprehensive definition of NSSI in the last 10 to 15 years; this indicates that historically, knowledge about NSSI was limited and just started to develop [26].

Despite these shortcomings, some dedicated psychological questionnaires help to identify individuals who are currently involved in NSSI (see Measures). However, it is shown that due to some latent relations among the questions, the combination of them describes involvement in NSSI better than using them separately [27]. One of the limitations the questionnaires convey is the numerous questions that a person must answer to be diagnosed. Answering a large number of questions is clearly very time-consuming and tedious so the results are likely inaccurate due to the frustration of the participants while answering them [27]. Therefore, many people answer them carelessly or inattentively. One reason that we still have to stick to the questionnaires is that some injurers hide their behaviors so that they are not objected to [7]. Therefore, if we are to identify such injurers without any questionnaires, we can only do so if they are caught injuring or they make blunders.

Hence, it is needed that academia extracts smaller subset(s) of the original questionnaire to 1) not let the participants become tired so that the answers tend to be more consistent, 2) omit the questions that explicitly ask about the participants’ involvement with NSSI so that participants will not answer with lies, and 3) omit the questions that may shift the attention of psychologists from actual facts about the participants’ involvement with NSSI to less important ones.

Traditionally, psychologists subjectively related one’s lifestyle and mentality to their involvement with NSSI, whereas Artificial Intelligence (AI)-based approaches hold promising results while noticing NSSI involvement [28]. Furthermore, AI approaches enable us to simultaneously consider multiple different factors, such as age, gender, etc., at the same time [29]. AI and machine learning fields of study try to enable computers to mimic living species’ neural systems so that they can also perceive and/or behave as living species do [30–32]. For example, an artificial spider walking like a real one [33], or an AI-based model locating cancer nuclei on histopathology images like an expert does [34].

AI-based diagnosis methods have seen particular attention in medical and healthcare systems as they can often become a life-saving tool for people [34–40]. For example, since data from Alzheimer’s Disease (AD) are multimodal and time series in nature, it is a burden for specialists to handle all the data that their patients provide; thus, they may not be able to correctly decide on the outcomes [38]. Hence, state-of-the-art AI-backed approaches can be utilized to predict AD progression 2.5 years in the future, based on the multimodal time-series data [38]. In another related work, a mobile application was introduced to detect skin disease via AI [39]. The method used MobileNet-V2 and Long Short-Term Memory (LSTM) to make an algorithm that could understand complex patterns on skin and maintain stateful information for precise predictions.

As a combination of wearable sensors and social networking, a healthcare monitoring framework based on the cloud environment and a big data analytics engine is available that stores and analyzes healthcare data provided by sensors or social networks [40]. The system classifies the healthcare data to predict drug side effects and abnormal conditions in patients. That also classifies patients’ health conditions using their healthcare data that are related to diabetes, blood pressure, mental health, and drug reviews. In another similar work, Bluetooth Low Energy (BLE)-based sensors are to gather users’ vital signs data such as blood pressure, heart rate, weight, as well as blood glucose and to send them to smartphones. Then, AI-based methods are to provide early prediction of diabetes given the user’s sensor data as input [37].

Therefore, healthcare data classification via AI provides the quickest and the most accurate automated approaches for medical centers as well as in-home diagnosis systems. These methods remove the subjectivity of specialists and try to understand and map the input values to unbiased outputs [35]. In the current case, for instance, similar methods can input data representing people’s behaviors, and decide whether they are involved in NSSI or not [28]. These methods could potentially mitigate some of the issues described earlier and can be delivered relatively quickly.

Considering these, for the first time, we aimed to summarize 17 different questionnaires (of 662 questions in total) to only 22 questions (items) using feature selection and data mining techniques. Then, we build an AI approach that classifies people with either NSSI positive or negative with 83.6% accuracy by deciding on the answers to these questions. The model can be used as a decision support system (DSS) to help psychologists quickly identify their patients with acceptable accuracy. In summary, this paper has five aspects of novelty:

- Reduces the 667-item questionnaire for NSSI identification to only a 22-item one.
- Proposes the fastest approach for identifying people involved in NSSI via Machine Learning and data mining techniques.
- Validates the abstracted questionnaire via two different statistical populations.
- Gives insight into utilizing Data Mining methods to abstract large questionnaires.
- Provides a public dataset for future studies in similar cases.

The remainder of the paper is organized as follows: information about participants, measures, and data preparation, as well as the utilized machine learning and data mining methods are available in Materials and methods. The results of the feature selection, abstracted questionnaire, and validation are available in Results, while the corresponding discussions are available in the Discussion section. Finally, to conclude the paper, the Conclusion section is available to summarize the paper and mention a limitation and a prospect.

## Materials and methods

### Participants

The present study builds on the research conducted by Jann MacIsaac [27]. Their participants were students of the University of Windsor, Canada, recruited by Jann MacIsaac. Their data collection process began in September 2017 and ended in April 2018. Initially, 314 participants were selected for their study, but 277 participants actually were included in the final dataset. Among them, 194 people did not have NSSI disorder, 44 were NSSI-Distal, and 39 were NSSI-Proximal. All of these 277 participants’ data is used in the current feature selection phase to choose the questions which are highly related to NSSI. Note that the dataset by Jann MacIsaac was not publicly available at the time of conducting this research (2019–2022). Thus, further information about it is not provided. Please contact them for more information.

After selecting features (questions) from the provided data in the first phase, an online website is created to test and validate the chosen features. Thus, 409 different participants from Azarbaijan Shahid Madani University, Tabriz, Iran are recruited. They were familiarized with the procedure in a virtual meeting and verbally agreed to participate (all data are kept anonymous with no traceable information. Those who did not consent to participate, did not provide any data; hence, the need for documented consent was not required as participants could deny participation if they did not consent to participate). All procedures performed in this study are in accordance with the ethical standards of the Institutional Review Board (IRB) of Azarbaijan Shahid Madani University and are reviewed and approved before the study began. After filtering the careless and inattentive participants, data from 245 participants is included in the second phase, whose ages vary from 18 to 35 (M = 27.88, SD = 9.26). The sample consisted of 152 females (62.04%) and 93 males (37.96%), whose demographics are presented in Table 1.

**Table 1:** Participants’ information regarding age, education, and NSSI behaviors.

Term	Number of participants by specific subgroups				
Participants	245				
Age	18 to 35				
Education	Diploma	Higher education	Bachelor’s	Master’s	Doctorate
	37	13	102	80	13
NSSI classes	Positive		Negative		
	Distal	Proximal	204		
	29	12			

### Measures

Jann MacIsaac included 19 questionnaires in their datasets, descriptions of which are as follows:

- **Demographic questionnaire:** The demographic information of all participants including age, gender, race/ethnicity, marital status, university year of enrollment, faculty, employment status, current residence, GPA, and meditation.
- **Deliberate Self-Harm Inventory (DSHI):** A 16-item self-report questionnaire to assess whether participants have ever performed a particular self-injurious behavior or not [6].
- **Inventory of Statements About Self-Injury (ISAS):** A two-part questionnaire, the first section of which assesses the lifetime frequency of 12 NSSI behaviors performed intentionally (i.e., on purpose) and without suicidal intent. Its second section assesses 13 potential functions of NSSI: affect-regulation, anti-dissociation, anti-suicide, autonomy, interpersonal boundaries, interpersonal influence, marking distress, peer-bonding, self-care, self-punishment, revenge, sensation seeking, and toughness [41].
- **Risk-Taking 18 (RT-18):** An 18-item questionnaire that is used to assess adults’ overall risky behaviors. This questionnaire has two levels: risk-taking and risk assessment. RT-18 sums all of the answers of a patient; the higher they score, the higher their level of risk-taking or risk assessment [42].
- **Difficulties in Emotion Regulation Scale 18 (DERS-18):** An 18-item questionnaire designed to assess clinical problems related to emotion regulation. The questionnaire consists of six main subgroups: awareness of personal emotions, transparency about personal emotions, acceptance of personal emotions, access to emotion regulation strategies, ability to participate in purposeful behaviors when exposed to negative emotions, and ability to manage impulses generated during negative emotions [43].
- **Toronto Alexithymia Scale 20 (TAS-20):** A 20-item questionnaire that assesses difficulty identifying and describing between feelings and bodily sensation, difficulty describing feelings, reduced daydreaming, and externally-oriented thinking [44].
- **Positive and Negative Affect Schedule (PANAS):** A 20-item self-report questionnaire designed to assess both positive affect and negative affect moods [45].
- **UPPS-P Impulsive Behavior Scale (UPPS-P):** A 59-item questionnaire that measures five distinct facets of impulsivity: negative urgency, positive urgency, sensation seeking, lack of perseveration, and lack of premeditation [46, 47].
- **NIH Flanker Inhibitory Control and Attention Test:** Assesses participants’ executive function and attention [27].
- **Mindful Attention and Awareness Scale (MAAS):** A 15-item questionnaire that assesses individual differences in the frequency of mindful states over time [48].
- **Patient Health Questionnaire-9 (PHQ-9):** A nine-item questionnaire that assesses participants’ depression [49].
- **Generalized Anxiety Disorder-7 (GAD-7):** A seven-item questionnaire that assesses participants’ general anxiety [50].
- **Perceived Stress Scale (PSS):** A 14-item questionnaire that assesses the level of tolerance of individuals (as a representative of perceived stress), when they are in a stressful situation [51].
- **Big Five Inventory (BFI):** A 44-item questionnaire that examines a person’s personality in five aspects: agreeableness, conscientiousness, extraversion, openness, and neuroticism [52].
- **Connor-Davidson Resilience Scale-25 (CD-RISC-25):** A 25-item questionnaire that assesses participants’ ability to deal with stress and adversity [53].
- **Social Provision Scale (SPS):** A 24-item questionnaire that assesses participants’ level of social understanding. It describes six different social functions or provisions that may be received from relationships with others: guidance, reliable alliances, reassurance of worth, attachment, social integration, and the opportunity for nurturance [54].
- **Inventory of College Students’ Recent Life Experiences (ICSRLE):** A 49-item questionnaire that assesses participants’ recent stressful events [55].
- **Self-Compassion Scale Short-Form (SCS-SF):** A 12-item self-report questionnaire that assesses participants’ feelings of self-compassion [56].

The datasets also contain validation questions (i.e., reversed items) to test the accuracy of the participants’ answers. The total number of available features (questions) is 662.

### Data filtering and pre-processing

In the original dataset, the optional questions, descriptive questions (i.e., “how is your hometown”), and the non-quantitative ones (such as those of NIH) are discarded. This way, the 662 features were reduced to 403. The analyses for the second phase of the test included filtering the outliers and calculating IRV (intra-individual response variability) index [57] across the 28 items (including the reversed items; see Validation and building a DSS model) to which participants responded. All of the analyses are performed using Python and libraries such as Pandas (https://pandas.pydata.org), SciPy (https://scipy.org), Matplotlib (https://matplotlib.org), Pingouin (https://pingouin-stats.org), and Seaborn (https://seaborn.pydata.org). Respondents with very low IRV values were excluded from analyses. Additionally, we excluded the participants whose differences between the reversed items and their original analogies were more than or equal to two. Lastly, we excluded any participants that took more than 20 or less than five minutes to complete the whole experiment, as our piloting suggested that the average time to complete the test was approximately 10 minutes.

### Feature selection and supervisor algorithms

The popular evolutionary algorithms, such as Genetics, Ant Colony (AC), and Particle Swarm (PS) algorithms as well as Linear Support Vector Classifier (LSVC), are used to select features [58] while nine machine learning algorithms, random forests (RF), support vector machine (SVM) with regularization parameter (C) = 1.1, decision tree (DT), k-nearest neighbor (kNN), perceptron, multilayer perceptron (MLP) with 10 hidden layers, the neurons of which are 80% more than the input size, linear discriminant analysis (LDA), AdaBoost (AB), and Long-Short Term Memory (LSTM) network, were to supervise them. Other default parameters are not changed. Lastly, the output neurons of each model conform to the number of classes (see Feature selection for more information).

The former algorithms (feature selectors) select varying subsets of questions in each epoch and have them learned by the latter algorithms. Supervisors (latter algorithms) are trained on the answers to those questions learning to map them to a particular NSSI class. It is clear that if the supervisors achieve acceptable evaluation results on a particular subset, the items in the subset highly correlate with NSSI; and thus, can be excellent representatives of NSSI involvement [58].

The LSTM model has four memory layers with 22 inputs and 22 outputs each, followed by three fully-connected layers with 128, 256, and 64 neurons in their hidden layers. Its last layer has two or three neurons (based on the output classes; see Feature selection for more information). Except for the last layer which has the Soft-Max activation function, others have ReLU.

### Evaluation metrics

To evaluate the efficiency of the selected features, the Precision, Recall, F1-score, and Accuracy metrics are used, which are extracted as follows [59, 60]:

$$Recall_{i}=\frac{TP_{i}}{TP_{i}+FN_{i}}$$ (1)

$$Recall=\frac{1}{\left|C\right|}\sum_{i=1}^{\left|C\right|}\frac{TP_{i}}{TP_{i}+FN_{i}}=\frac{\sum_{i=1}^{\left|C\right|}{Recall}_{i}}{\left|C\right|}$$ (2)

$$Accuracy=\frac{\sum_{i=1}^{\left|C\right|}\frac{TP_{i}+TN_{i}}{TP_{i}+{FN}_{i}+FP_{i}+{TN}_{i}}}{\left|C\right|}$$ (3)

$$Precision_{i}=\frac{TP_{i}}{TP_{i}+FP_{i}}$$ (4)

$$Precision=\frac{1}{\left|C\right|}\sum_{i=1}^{\left|C\right|}\frac{TP_{i}}{TP_{i}+FP_{i}}=\frac{\sum_{i=1}^{\left|C\right|}{Precision}_{i}}{\left|C\right|}$$ (5)

$$F_{1}=2\times \frac{Precision\times Recall}{Precision+Recall}$$ (6)

In the above relations, |*C*| represents the total classes, which in our case, either *C* = {No NSSI, NSSI Distal, NSSI Proximal} or *C* = {NSSI Neg., NSSI Pos.} (see Feature selection for more information). *i* shows the class, the metrics of which are being calculated. *TP*_*i*_ indicates the number of cases that belong to the *i*^th^ class and are classified correctly, *TN*_*i*_ indicates the number of cases that do not belong to *i*^th^ class and were not classified as class *i* either. In contrast, *FP*_*i*_ indicates the number of cases that did not belong to class *i* but were incorrectly classified as class *i*, and finally, *FN*_*i*_ indicates the number of cases that belong to class *i* but were classified incorrectly. Precision shows how much we can rely on the model when it classifies participants, while recall measures the ability of the model to find all the correct cases in a given dataset. Finally, the F1-score is the weighted harmonic mean of the precision and recall, and it is beneficial while finding the best trade-off between the two quantities [61]. Note that as the approach is a classification problem, statistical tests do not increase the analytical deduction of the study as the lists being tested are comprised of only two or three different discrete numbers. Statistical tests are informative in regression problems that the values are continuous. Hence, the values in the current task can never meet normality (as required by the Central Limit Theorem) to be evaluated via statistical tests.

## Results

### Feature selection

First, we tested our approach on three classes (No NSSI, NSSI Distal, and NSSI Proximal) without and with data augmentation. We initially tested a different number of features (questions), but only the best and second-best performing ones are reported in Table 2. Also, among the mentioned feature selectors, only Genetics and LSVC had promising results; therefore, others are omitted from comparisons. As it is evident, the DT with the genetics algorithm obtained the highest overall efficiency whose accuracy, precision, recall, and F1-score are 58.9%, 50%, 47.5%, and 48.7% respectively. To improve the results, we augmented data using SMOTE synthetic method [62] then selected features, and illustrated its results in Table 3. In this case, the most efficient result was that of the genetics algorithm with an SVM model, the accuracy, precision, recall, and F1-score of which are 71.4%, 55.1%, 56.5%, and 55.7% respectively. According to the F1-scores, all of these approaches are behaving randomly and are not reliable at all.

**Table 2:** Comparison of the results of the algorithms on the initial dataset and the ones with feature selection approach without data augmentation (no. class = 3).

Algorithm	Original dataset (403 features)				Feature selection							
					LSVC (25 features)				Genetics (28 features)			
	A*	P*	R*	F*	A*	P*	R*	F*	A*	P*	R*	F*
RF	**69.6**	33.3	23.2	27.3	69.6	33.3	23.2	27.3	62.5	29.9	22.4	27.0
SVM	67.9	41.5	48.2	44.6	69.6	33.3	33.5	**33.4**	69.6	33.3	23.2	27.3
DT	60.7	38.1	39.0	38.5	55.4	29.8	**38.0**	27.3	58.9	**50.0**	**47.5**	**48.7**
kNN	69.6	33.3	23.2	27.3	69.6	33.3	23.2	31.8	**69.6**	33.3	23.2	27.3
Perceptron	67.9	41.5	**53.8**	**46.9**	39.3	35.4	28.8	31.8	69.6	33.3	23.2	27.3
MLP	64.3	**42.6**	51.5	46.6	55.4	**35.5**	28.8	31.6	67.9	35.3	40.4	37.7
LDA	58.9	42.5	39.1	40.7	60.7	32.4	30.8	27.3	69.6	36.2	56.8	44.2
AB	49.7	48.3	43.1	45.5	35.1	24.9	36.2	29.4	43.6	44.5	48.2	46.2
LSTM	69.3	44.2	38.6	41.21	**70.0**	34.8	29.2	31.7	68.4	49.8	44.0	46.7

A = Accuracy (%), P = Precision (%), R = Recall (%), F = F1-score (%)

**Table 3:** Comparison of the performances of LSVC and the genetics algorithm on the augmented dataset (no. class = 3).

Algorithm	LSVC (47 features)				Genetics (75 features)			
	A	P	R	F	A	P	R	F
RF	**69.6**	33.3	23.2	27.3	67.9	32.5	23.0	26.9
SVM	58.9	**46.7**	**45.5**	**46.1**	**71.4**	**55.1**	**56.5**	**55.8**
DT	53.6	38.0	38.7	38.3	53.6	32.3	31.7	32.0
kNN	30.4	35.9	36.7	36.3	23.2	41.5	32.9	36.7
Perceptron	64.3	39.8	41.7	40.7	46.4	53.5	47.9	50.5
MLP	60.7	31.9	30.1	31.0	58.9	46.2	45.0	45.6
LDA	62.5	45.5	44.8	45.1	51.8	49.4	45.2	47.2
AB	46.4	46.3	42.9	42.6	38.1	44.2	41.2	42.6
LSTM	61.3	44.2	44.9	44.5	66.7	52.6	49.1	50.7

To overcome the random behavior, the three classes are reduced to only two, being NSSI Positive (the combination of NSSI Distal and NSSI Proximal) and NSSI Negative. Note that the results of future experiments are only reported based on the genetics algorithm since it was more accurate than LSVC (and others). Table 4 compares the models’ efficiencies considering the initial features and selected features. As is evident, RF obtained the highest efficiency with an accuracy, precision, recall, and F1-score of 80.0%, 73.1%, 76.5%, and 74.8% respectively. To see how data augmentation methods contribute to the accuracy, Table 5 compares the effects of ADASYN, SVM-SMOTE, and SMOTE data augmentation methods, all of which led the feature selector to select 45, 34, and 19 questions respectively. It is evident from the table that ADASYN with DT achieved an accuracy, precision, recall, and F1-score of 81.6%, 81.0%, 80.2%, and 80.6% respectively, whereas those of SVM-SMOTE with LDA were 76.2%, 75.8%, 73.2%, 74.5% respectively; those of SMOTE with LDA were 71.4%, 71.2%, 68.5%, and 69.8%.

**Table 4:** Comparison of the performance of the algorithms on the initial dataset and the one with features selection (no. class = 2).

Algorithm	Initial dataset (403 features)				Feature selection			
					Genetics (22 Features)			
	A	P	R	F	A	P	R	F
RF	69.6	50.0	34.8	42.3	**80.0**	**73.1**	**76.5**	**74.8**
SVM	69.6	50.0	34.8	42.3	74.6	67.3	69.8	68.5
DT	66.1	60.7	60.4	60.5	65.7	59.2	59.9	59.5
kNN	69.6	50.0	34.8	41.3	70.1	55.8	54.7	55.2
Perceptron	71.4	52.9	85.5	65.3	60.4	58.7	58.0	58.3
MLP	**75.0**	**70.4**	**70.4**	**70.4**	74.6	70.4	72.0	71.1
LDA	67.9	65.3	63.8	64.5	72.9	62.0	62.4	62.2
AB	60.7	53.5	53.5	53.5	69.1	68.7	68.4	68.5
LSTM	74.7	68.3	69.9	69.0	72.6	67.4	68.8	68.0

**Table 5:** Comparison of the effect of the three data augmentation methods on the algorithms with selected features (no. classes = 2).

Algorithm	SMOTE (19 features)				SVM-SMOTE (34 features)				ADASYN (45 features)			
	A	P	R	F	A	P	R	F	A	P	R	F
RF	69.6	60.0	62.5	61.2	70.1	54.8	55.7	55.2	74.4	64.6	70.0	67.2
SVM	69.6	68.3	66.2	67.2	73.0	70.1	71.3	71.7	76.2	76.5	73.0	74.7
DT	57.1	52.6	52.4	52.4	52.2	51.2	50.8	51.0	**81.6**	**81.0**	**80.2**	**80.6**
kNN	67.9	70.3	67.2	68.7	41.5	44.2	45.3	44.7	41.5	53.2	55.2	54.2
Perceptron	69.6	55.0	61.0	57.8	71.4	65.9	68.3	67.1	71.9	56.7	60.1	58.4
MLP	64.3	59.4	58.9	59.1	65.5	68.1	64.2	66.1	79.3	78.2	78.2	78.2
LDA	**71.4**	**71.2**	**68.5**	**69.8**	**76.2**	**75.8**	**73.2**	**74.5**	71.9	71.6	68.9	70.2
AB	69.6	66.6	65.3	65.9	65.5	60.5	60.3	60.4	70.1	64.0	64.4	64.2
LSTM	68.4	67.0	64.5	65.7	73.2	74.7	72.1	73.3	77.9	75.2	74.8	74.9

### The abstracted questions

According to the results in Tables 4 and 5, it is possible to reduce the original questionnaire to a 19- or 22-item one. As the latter showed a better performance compared to the former, we selected the latter as our abstracted questionnaire. Although the former has fewer items, the difference (three questions) is not significant enough to lead the participants to answer carelessly. Hence, we chose performance over conciseness. Although there are statistical ways to see which questionnaire is better, since machine learning algorithms can consider relations between items better than the statistical methods, we opted for the modern (machine learning) solution for this task and thus relied on the provided evaluation metrics. The items in the abstracted questionnaire are selected from nine questionnaires: Demographic Information Questionnaire, TAS [44], PANAS [45], UPPS-P [46, 47], PSS [51], CD-RISC [53], SPS [54], ICSRLE [55], SCS-SF [56]; and are available in Table 6. Although the set of 45 items better represents NSSI behaviors (according to Table 5), its participants may still respond carelessly due to 45 tedious questions; hence this one is also discarded. Despite showing a 74.8% F1-score, the 22 items are still more accurate than a subjective psychologist who is exposed to fatigue and has to assess many participants who might not be honest with their responses in a large and tedious questionnaire.

**Table 6:** Questions of the abstracted dataset.

Item	Question	From questionnaire	Original index	Value
1	Academic year	Demographic	-	Integer [0, 5] (0 = Not a student)
2	Gender	Demographic	-	Integer [0, 1] (1 = Female; 0 = Male)
3	It is difficult for me to find the right words for my feelings	TAS-20	2	Integer [1, 5]
4	I prefer to just let things happen rather than to understand why they turned out that way	TAS-20	8	Integer [1, 5]
5	People tell me to describe my feelings more	TAS-20	12	Integer [1 5]
6	When things look hopeless, I do not give up	CD-RISC	12	Integer [1, 5]
7	I think of myself as a strong person*	CD-RISC	17	Integer [1, 5]
8	I have a strong sense of purpose	CD-RISC	21	Integer [1, 5]
9	I have some decisions about (an) intimate relationship(s)*	ICSRLE	17	Integer [1, 5]
10	I am dissatisfied with my physical appearance	ICSRLE	45	Integer [1, 5]
11	I find the course(s) uninteresting	ICSRLE	46	Integer [1, 5]
12	When I am feeling down, I tend to feel like most other people are probably happier than I am	SCS-SF	4	Integer [1, 5]
13	I am intolerant and impatient toward the aspects of my personality that I do not like	SCS-SF	12	Integer [1, 5]
14	I have often dealt successfully with irritating life hassles in the last month*	PSS	4	Integer [1, 5]^R^
15	No one needs me to take care of them	SPS	24	Integer [1, 5]^R^
16	I feel I am guilty*	PANAS	6	Integer [1, 5]
17	I am a determined person*	PANAS	15	Integer [1, 5]
18	I am an attentive person*	PANAS	16	Integer [1, 5]
19	I will try anything once	UPPS-P	8	Integer [1, 5]^R^
20	I quite enjoy taking risks	UPPS-P	23	Integer [1, 5]^R^
21	When I am upset, I often act without thinking	UPPS-P	29	Integer [1, 5]^R^
22	Others are shocked or worried about the things I do when I am feeling very excited	UPPS-P	35	Integer [1, 5]^R^

*These questions have been revised (minor) to semantically match with other questions.

^R^These questions were also included in their reverse form in the validation phase (see Validation and building a DSS model).

Since the previous methods divided data to test splits to select features, it is not clear whether the algorithms are under- or over-fitted. Therefore, we used 5-fold cross-validation on the 22 questions with the three best-performing algorithms (also with and without data augmentation) to assure consistency and presented the results in Table 7. Evident from the table is, the results are consistent with the previous table (particularly Table 4) as the difference between them is marginal. That is, the best-performing algorithm is still RF whose evaluation results are similar to the previous table that indicates the significant consistency of the selected features.

**Table 7:** Results of the three-best performing machine learning algorithm on the abstracted questionnaire (no. class = 2; no. items = 22).

Algorithm	Data augmentation	Avg. Accuracy (%)	Avg. Precision (%)	Avg. Recall (%)	Avg. F1-score (%)
**RF**	-	**80.5**	**74.0**	**75.7**	**74.8**
SVM		75.2	67.5	67.3	67.4
MLP		74.8	71.0	72.4	71.7
**RF**	SMOTE	***81*.*2***	***73*.*8***	***75*.*2***	***74*.*5***
SVM		67.9	66.3	65.8	66.0
MLP		78.6	72.5	76.4	74.4
RF	SVM-SMOTE	***80*.*9***	72.5	74.3	73.4
SVM		78.6	68.0	64.5	66.2
**MLP**		75.2	***73*.*8***	***74*.*4***	***74*.*1***
**RF**	kNN-SMOTE	***81*.*4***	***75*.*3***	***72*.*2***	***73*.*7***
SVM		77.3	67.2	65.4	66.3
MLP		75.2	70.8	69.2	70.0
**RF**	ADASYN	***80*.*5***	***73*.*9***	***74*.*0***	***73*.*9***
SVM		73.6	66.3	66.7	66.5
MLP		76.8	73.1	73.1	73.1

### Validation and building a DSS model

To validate the abstracted questionnaire, we designed an online website to ask participants to provide data. The website had a description section to make participants familiar with its questionnaire and its aims. We added six reversed items between the abstracted 22 items to assure the answers’ consistency and to be able to filter careless participants. Finally, the Cronbach alpha of the pruned responses is 0.82.

**Fig 1:**
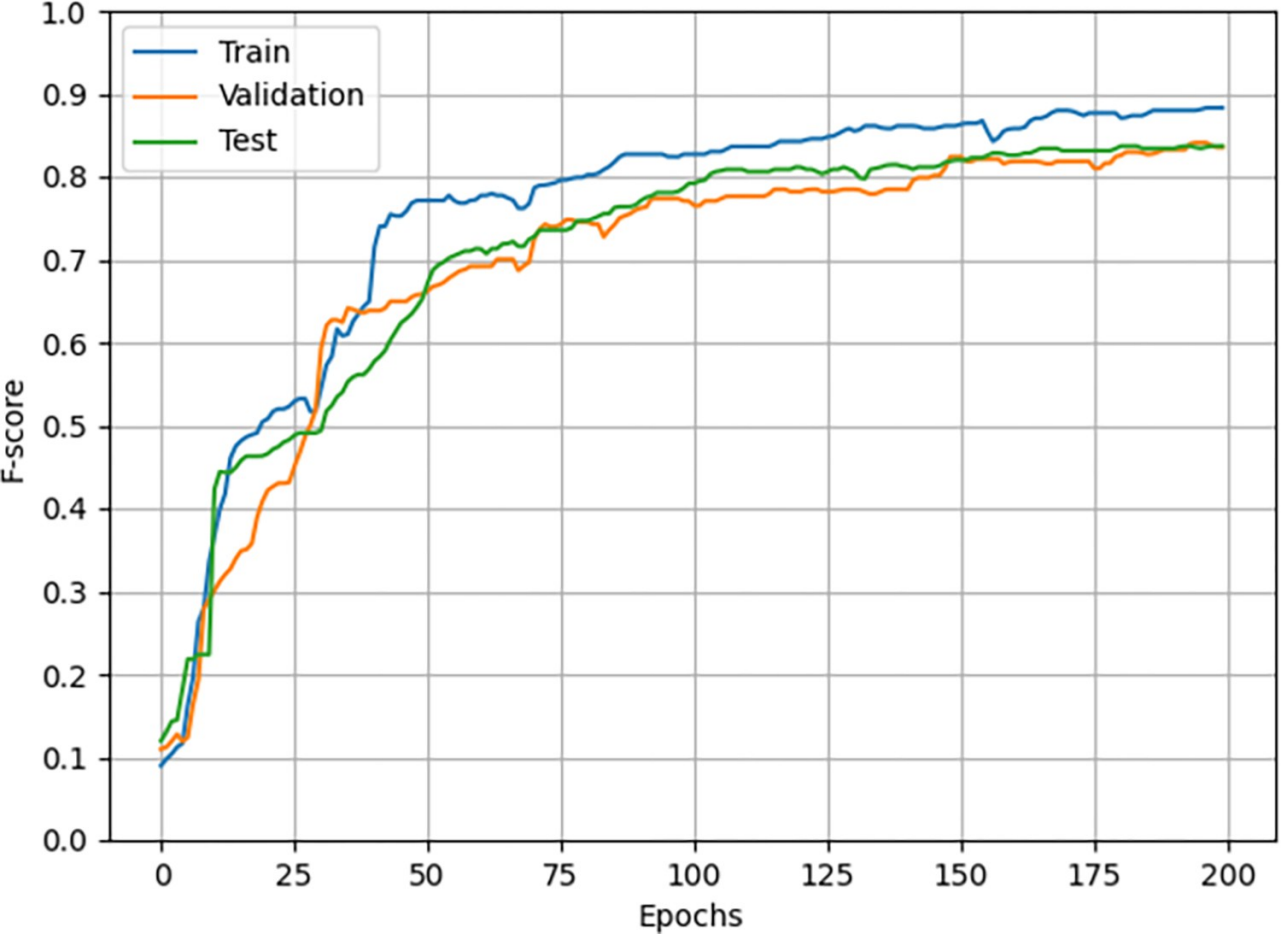
F1-score fluctuations of the MLP model per epoch.

**Fig 2:**
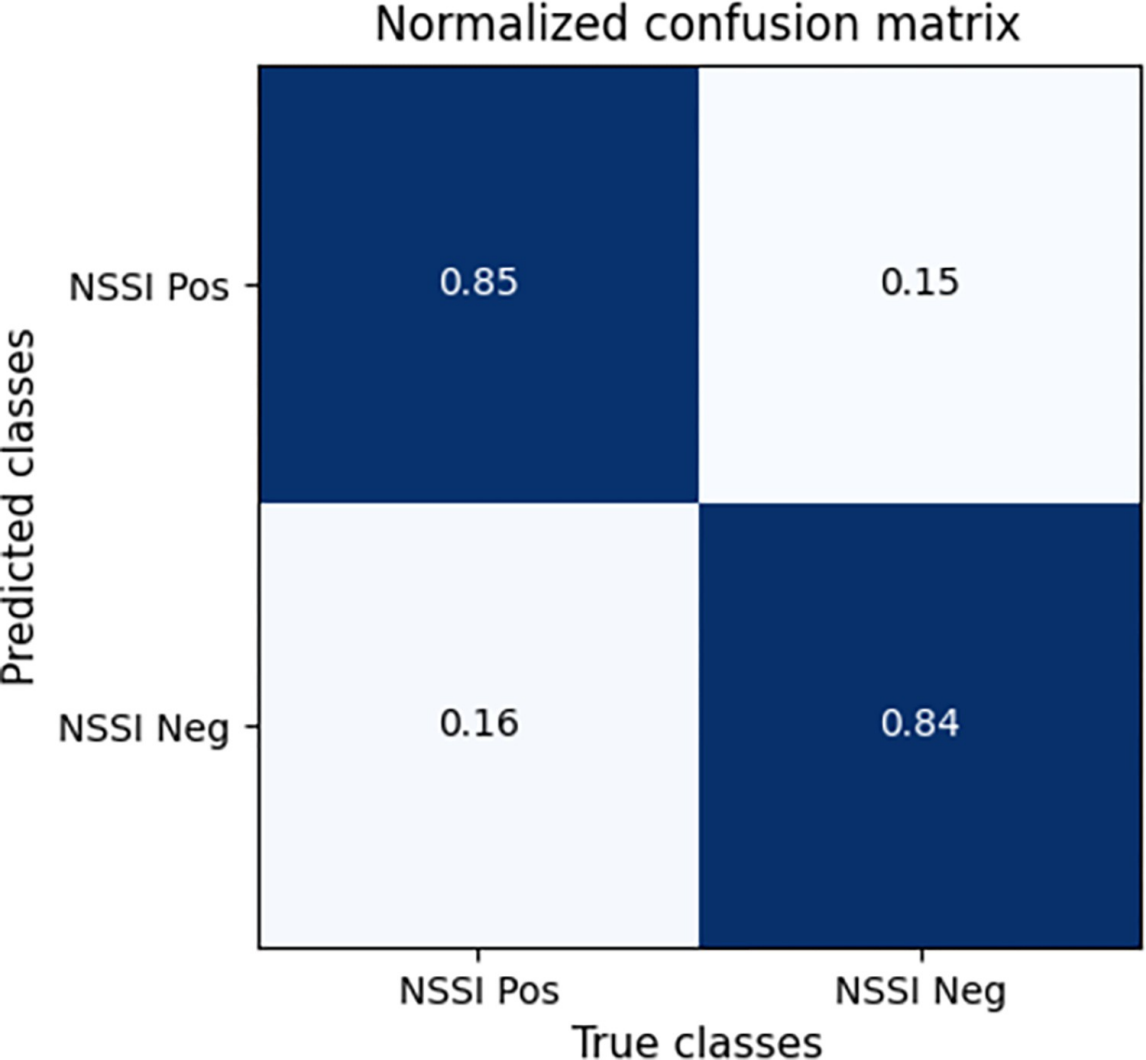
Normalized confusion matrix of the MLP model.

Based on the pruned data, we train eight machine learning models which are to classify participants into two classes (NSSI Positive and NSSI Negative) based on their scores (answers) to each question. The reversed items are not used in this phase to see how well the 22 items can represent NSSI involvement. The performances of the models are available in Table 8 and are calculated using 5-fold cross-validation. Note that we tested the four data augmentation algorithms for each of the methods in Table 8; however, only the best-performing one is reported per each algorithm. It is evident that overall, its results are consistent with those in Tables 4 and 7 since the three tables have negligible differences from each other. This endorses that the selected features are consistence in both statistic populations (the initial dataset and the collected one) and can represent NSSI involvement among different nations. However, it was interesting to see the MLP algorithm could outperform its previous model (that in Table 7) and rank first with an accuracy, precision, recall, and F1-score of 83.6%, 83.6%, 83.9%, and 83.7% respectively. The MLP model has 10 hidden layers and 40 neurons in each layer. Moreover, the RF algorithm shows a very narrow difference from that in Table 7, indicating its overall stability through different datasets. Since MLP outperformed other methods, only its results are visualized in the following. Thus, Fig 1 shows average F1-score fluctuations per epoch in the 5-fold cross-validation, while Fig 2 depicts the normalized average confusion matrix of the MLP model in the same setting.

**Table 8:** Results of the trained models on the abstracted questionnaire.

Algorithm	Data augmentation	Avg. Accuracy (%)	Avg. Precision (%)	Avg. Recall (%)	Avg. F1-score (%)
RF	-	*81*.*2*	*75*.*5*	*73*.*7*	*74*.*6*
SVM	ADASYN	80.1	67.2	68.4	67.8
DT	-	62.2	61.5	61.2	61.3
kNN	ADASYN	73.4	58.1	54.2	56.1
Perceptron	ADASYN	59.5	58.1	56.2	57.1
MLP	SVM-SMOTE	**83.6**	**83.6**	**83.9**	**83.7**
LDA	-	73.1	62.0	62.4	62.2
AB	ADASYN	71.7	71.7	72.1	71.9
LSTM	ADASYN	74.6	69.8	70.2	69.9

## Discussion

NSSI is a psychological disorder that a person intentionally harms themselves without intending to die [1]. In the present study, an attempt has been made to provide a simpler and more efficient method for predicting NSSI disorder using several basic classification algorithms. For this purpose, after conducting various experiments using data mining and feature selection, an abstracted questionnaire was extracted which is comprised of only 22 questions, only three percent of the initial 662 questions, and an abstract of 17 different questionnaires. Answering these questions only takes about five to six minutes (without including reversed items) which potentially increases confidence and accuracy since participants are not bored while answering. Consequently, a machine learning algorithm can classify a participant with 83.6% accuracy. Our study lets psychologists find people involved in NSSI and start psychotherapeutic treatments as soon as possible. Another convincing reason for considering this study worldwide would be its independence on the questions that potentially could be answered with lies. Some paramount examples of such questions would be those asking participants’ involvement in deliberate self-injuring. The majority of people who are involved in NSSI are shown to be hiding this trait as they wish not to be objected to. Hence, the inclusion of such questions may alter the results and thus, they had better not be included.

Here, we mainly used traditional machine learning methods instead of more complex deep learning-based ones since the former methods are shown to be more robust when they are to consider statistical aspects or relations between different components of the input data on the one hand, and can perform better than the latter approaches when the data is sparse and the inter-item variety is not significant on the other hand [53–60]. Additionally, the used data in this approach are self-explanatory and do not need feature extraction phase(s). Therefore, there is no necessity to use deep learning methods as they usually rely on larger datasets for better performance. However, since the questions were shown to the participants in a static order, we thought that using an LSTM could be beneficial as it can relate the bias of previous questions to the answer of the current one. Yet, results suggested that it was not the case in this approach. We did not also use CNNs because they are inspired by neural behaviors in the eyes’ receptive field and are usually well-performed on visual tasks, such as image detection, image classification, etc. [63]. Additionally, they multiply adjacent values of the input to form filters; thus, the importance of the values will be diminished if used in cases like the one in the present study [63].

As seen in Tables 2 and 4, the inclusion of the whole dataset even confuses a machine learning method. Therefore, psychologists, who are exposed to heavy workloads and often fatigue, are not immune to the confusion and thus, may decide subjectively. However, using feature selection and machine learning algorithms, we proved that the inclusion of a more refined set of questions can be less confusing and aid people in making more accurate decisions. Although some may say that the achieved level of accuracy in the proposed research is still far from an accurate decision made by a psychologist, considering the delivery time and convenience of the provided abstracted questionnaire, it suffices the needs of the current state of psychological expectations. Furthermore, it is still more precise than a subjective psychologist who is exposed to fatigue and has to assess many participants who might not be honest with their responses in (a) large and tedious questionnaire(s).

As supported by other studies, distress (Items 3, 6, 7, 8, 12, 14, 20) [64–69], depression and substance use disorder (SUD) (Items 3, 4, 5, 8, 9, 12, 15, 18, 19, 20, 21) [1, 67, 70–72] are strong predictors of NSSI. Despite existing in questionnaires previously, Emotional Reactivity (ER) has been recently proven to be a contributing factor for NSSI identification (Items 12, 16, 21, 22) [73]. Dissatisfaction with one’s body and trauma (Items 10 and 13) [1, 5, 16, 17, 67, 74] as well as motivation and activity (Items 11, 17, 18) are shown to be correlated with NSSI [13, 75]. Gender (Item 2) is shown to be a strong factor contributing to NSSI and suicidal attempts among people [67, 72]. Age is another factor to be taken into consideration when identifying individuals with NSSI [3–7]; however, our feature selector found that in our dataset, the academic year correlates with NSSI better than the age factor (Item 1). Hence, mapping latent features of the ages to the academic years, our method used the latter data to predict NSSI involvement. All in all, it is evident that the chosen questions are backed by previous studies, all of which provide the grounds to increase reliance on the proposed abstracted questionnaire. Additionally, as mentioned, since the results were consistent among two different populations, psychologists can have more confidence when diagnosing patients using our method.

Three pioneering studies used data mining techniques to predict NSSI, two of which aimed to identify subgroups of individuals who engaged in NSSI by identifying splits on related variables [28]. The first used a machine learning technique to examine splits in the number of NSSI acts during the previous year as predicted by participant-reported psychological difficulties. Results demonstrated significant splits between zero and one-or-more past year NSSI acts on the one hand, and between five and six-or-more past year NSSI acts on the other hand. This suggested that participants reporting six or more past-year NSSI acts may represent a more severe group of self-injurers [1]. Another study built on the previous one and examined splits in NSSI behavior age of onset predicted by prior Self-Injurious Thoughts and Behaviors (SITB), including NSSI characteristics (i.e., number of NSSI-related hospital visits, NSSI frequency, etc.), suicidal ideation, suicidal planning, and suicidal attempts. Results suggested there was a potential subgroup in the data representing those with an earlier age of onset (i.e., approximately 12 or younger); this subgroup reported a greater number of NSSI methods, NSSI frequency, and NSSI-related hospital visits, in addition to an increased likelihood of having suicidal planning [71]. The final study employed two machine learning techniques to identify important indicators of NSSI frequency, both explaining a significant proportion of variance in NSSI frequency (R^2^ = 0.48 and 0.46, respectively). Models indicated that the number of NSSI methods was the most important indicator of lifetime NSSI frequency; after removing the number of methods from the models, suicidal planning and depressive symptoms emerged as the most important in the prediction of NSSI frequency [3].

Previously, state-of-the-art studies provided time-series analysis to determine whether a person is involved in NSSI or not. For instance, within a sample of 1,021 high-risk self-injurious and/or suicidal individuals, Huang et al. [76] examined the accuracy of three different complex model types in predicting NSSI across 3, 14, and 28 days. In another study, Marti-Puig et al. [77] built a mobile application to collect data so that later they could classify NSSI in young adults focusing on their emotions only. After the data collection phase, they used the data as a time series to test one’s involvement in NSSI. It is clear that such approaches require multiple records of data for their decision-making process, none of which are convenient when injurers are to be identified in only one and the first session of their therapies. Moreover, the former approach only achieves an 84.0% F1-score after processing data entries of the last three days and the latter achieves merely a 22.9% F1-score after processing the data of the last 15 days of a person. Additionally, they ask the user to clarify whether they have been involved in NSSI as the ground truth data. Yet, users may deliberately lie about this question in particular, if they do not want to be objected as mentioned previously. However, our method achieves an 83.7% F1-score after processing only 22 items without requiring the patient to clarify their NSSI behaviors. These automatically increase the reliance on our approach and make it one of the most convenient methods of identifying those involved in NSSI.

Some other successful machine learning-based methods are to provide insight into key factors when NSSI involvement is being decided by a psychologist. For example, Gradus et al. [72] developed gender-stratified classification trees and random forests using 1,458 predictors, including demographic factors, family histories, psychiatric and physical health diagnoses, surgery, and prescribed medications. They found that SUD, prescribed psychiatric medications, previous poisoning diagnoses, and stress disorders were important factors for predicting suicide attempts among men and women. Wallace et al. [67] in 2021 tested classification trees that evaluated 298 potential correlates of NSSI and suicidal ideation across self-identified women and men. Psychopathology, poorer psychological well-being, and other SITBs emerged as important correlates for all participants. Trauma, disordered eating, and heavy alcohol use were salient among women, whereas alcohol use norms were important correlates among men. In a similar study, Yang et al. [78] in 2022 proposed an SVM model which deduced adolescents’ gender, paranoid and histrionic personality traits, suffring physical abuse in childhood, emotional non-acceptance, and education level were associated with an increased risk of NSSI. These may alert psychologists to pay more attention to the mentioned factors. Nevertheless, solely outlining key factors may not be reasonable enough to make psychologists opt for such methods and decide on a diagnosis. Perhaps, using the factors all together to also classify the trait would be more acceptable among psychologists, which requires more complicated processes on data. Automated methods, on the other hand, can quickly do the computation to provide an outcome on the diagnosis. This also contributes to the convenience of such methods while saving some time and energy for the psychologist.

Although our baseline study is conducted on a relatively small statistical population without a broader validation, studies involving self-reports that lack additional objective components still suggest promising aspects of novelties for future research. For instance, predicting a marker that had previously not been researched extensively, Twivy et al. [79] found that differences in affective flexibility towards emotional stimuli may be a positive indicator of anxiety. Soroski et al. [12] built an online website to collect the voices of their participants to propose an Alzheimer’s detection system. Sanders and Nosofsky [11] crowdsourced participants to reconstruct psychological feature space for natural object detection in machine learning applications. That said, a need for a universal measure free from self-reporting constraints is demonstrated, both for the reliability and validity of current and future research. This can be investigated using such pilot studies which may lack additional objectiveness yet fillip the stagnant realms of research.

Currently, other means of determining human cognition and emotions are under review to act as possible alternatives to subjective self-reports. A report studying adults with schizophrenia spectrum disorders found important indicators in these models include duration of illness, number of hospitalizations, emotional and physical abuse in childhood, as well as drug usage or abuse [80]. Furthermore, computational models are concurrently being explored as a possible avenue to examine gender-specific risk profiles for suicide attempts potentially providing an objective lens through which to take into consideration SUD treatment, prescribed psychiatric medications, previous poisoning diagnoses, and stress disorders [72].

Future studies may aim to recruit non-university participants from other countries, although the strength of our study was the generalizability of the sample as we recruited NON-WEIRD (Western, Educated, Industrialized, Rich, and Democratic) [81] participants. Moreover, despite reducing the number of questions, the system still requires around five minutes (reversed items not included) of a person so that it can decide on a result. Therefore, in the future, other cognitive constructs could be used to make the NSSI classification more automated, such as via visual tasks (i.e., eye tracking, visual search, etc.).

## Conclusion

People who are involved in NSSI disorder tend to deliberately damage their body tissues to dispel the negative psychological effects of their lives. Since they may hide their behaviors, their parents or caretakers can only notice their behaviors if they catch the injurers while injuring themselves. Another alternative is via dedicated questionnaires. However, they are boring and open to errors and lies. Therefore, we abstracted an original questionnaire of 662 items to own only 22 questions via utilizing data mining techniques, and trained machine learning algorithms to classify participants into two classes of NSSI Positive and NSSI Negative with 83.6% accuracy. Since answering these questions only takes about five to six minutes, the accuracy and confidence will be automatically increased.

That said, the limitation of the current study is that it has been evaluated on only two statistical populations. Further evaluation requires recruiting more participants from different nations to examine the generalizability of such approaches. As a future study, the approach can be further expanded by cognitive models. That is, cognitive models may be able to map particular aspects of complex human behaviors (i.e., their walking style, pauses in their speech) to NSSI. Hence, to leverage Artificial Intelligence, academia may further examine such models for more ubiquity in different places.
